# Practical considerations in the selection of seed strength for prostate implants

**DOI:** 10.1120/jacmp.v16i5.4720

**Published:** 2015-09-08

**Authors:** Sarah L Elliott, Catherine L. Beaufort, Jeremy L. Millar

**Affiliations:** ^1^ Radiation Oncology AlfredHealth Melbourne Victoria Australia; ^2^ Department of Epidemiology and Preventive Medicine Monash University Melbourne Victoria Australia

**Keywords:** brachytherapy, prostate implants, I‐125, seed strength

## Abstract

There are advantages in using lower numbers of higher activity seeds for prostate seed implants. This work investigated the use of higher strength seeds for our manually optimized prostate implants. Following a planning study using a range of seeds strengths between 0.4 U and 0.7 U, a series of patients were implanted using seeds of strength ∼0.7U. Twenty consecutive patients were selected for this study; ten patients were implanted with 0.4 U seeds and the next ten patients implanted with 0.7 U seeds. Postimplant dosimetry for the target volume, urethra, and rectal wall was compared between the two groups. Our data showed a small and insignificant decrease in the total theatre time when implanting seeds of higher strength. The mean number of seeds required per implant decreased by over 30% for the 0.7 U implants, and the mean number of needles decreased by eight needles. The mean D90 (%) was marginally lower for the 0.7 U group, and spread over a wider range of values. Doses to the rectal wall were slightly higher for the 0.7 U group. At six years postimplant, the symptom scores for urinary and rectal toxicity and erectile function were similar to those reported before brachytherapy, with little differences between the 0.4 U and 0.7 U groups. Our experiences and practical advice in the selection of seed strength for prostate implants are reported in this paper.

PACS number: 87.53.Jw

## I. INTRODUCTION

Permanent seed implants (PSI) with radioactive I‐125 seeds have been used with increasing frequency in Australia for the curative treatment of prostate cancer, since the procedure was listed for Medicare support in late 2001. Since the inception of the William Buckland Radiotherapy Centre (WBRC) brachytherapy program in 1998, over 800 patients have been implanted with permanent radioactive I‐125 seeds for the treatment of early stage prostate cancer.

At the WBRC, a seed air‐kerma strength of just under 0.4 U (where $1U=1\mu {\text{Gym}}^{2}h^{-1}$) is currently used for PSI. This seed strength was initially selected for historical reasons; we implemented the Seattle planning method.^(1)^ This method follows the belief that, by using lower activity seeds, better dose homogeneity can be obtained and we would be less likely to overdose critical structures. On the other hand, there are advantages in using fewer but higher activity seeds, provided that clinical outcomes are equivalent. There are fewer total numbers of seeds and needles per implant, which reduces the overall cost of the seeds and the cost of theatre time, and fewer needles results in less tissue trauma to the patient caused by needle sticks.^(2)^

The optimum strength of I‐125 seeds for prostate PSI has been investigated by several groups, typically by comparing dosimetry between plans or implants using higher (0.5−0.8U) and lower (0.3−0.4U) seed strength.^3^, ^4^, ^5^, ^6^, ^7^, ^8^, ^9^ There is general agreement that satisfactory dosimetric target coverage can be achieved for a wide range of seed activities. However, the prevailing concern with implanting higher activity seeds is plan robustness, should there be seed placement error or seed migration. Studies simulating seed displacement and migration effects on dosimetric coverage and critical organ sparing as a function of seed strength have reported somewhat disparate conclusions. Some planning studies support higher seed strengths,^3^, ^4^ other studies prefer to plan with lower strength seeds,^5^, ^10^ while some planning studies suggest that the effect on dosimetry of perturbations on the plan is similar for high‐ and low‐strength seed arrangements.^2^, ^11^ It is evident that robust higher seed activity implants require carefully generated plans, with peripherally loaded seed patterns^12^, ^13^ and, where possible, from automated inverse planning methods.^2^, ^11^

Clinical studies at two centers have reported superior dosimetric prostate coverage with no significant dosimetric differences for implants using higher strength seeds in comparison to lower strength seeds.^6^, ^7^, ^8^, ^9^ Furthermore, recent publications have reported patient outcomes,^9^, ^14^ urinary toxicity,^8^, ^14^ and rectal toxicity^(14)^ up to ~ five years postimplant, with comparable results for high and low seed strength implants. However in the large study by Usamani et al.,^(9)^ a higher incidence of late rectal toxicities was noted in their higher seed strength cohort (>0.49U) of patients.

The aims of this study were to evaluate the use of higher activity seeds with our planning and implant technique, and to directly compare and report clinical data on urethral and rectal toxicity from such a study at six years postimplant. This paper reports a practical approach and considerations for a brachytherapy center in the selection of 0.4 U or 0.7 U seeds for prostate seed implants.

## II. MATERIALS AND METHODS

### A. Planning study

Twelve previously treated patients were selected according to prostate size, and replanned using seed strengths of 0.6 U or 0.7 U on Nucletron Plato Treatment Planning Software v 14.2.5 (Nucletron, Veenendaal, The Netherlands). Target volumes (the prostate plus a margin) were classified as small ($39-45{\text{cm}}^{3}$), medium ($55-61{\text{cm}}^{3}$) or large ($71-82{\text{cm}}^{3}$). For a simple and practical approach, our starting position for planning was to follow our standard planning guidelines; that is, using a modified Seattle approach and dosimetry limits for the target volume of D100: >0.49Gy; V100: >98%; V150: 52%−62%; and V200: 11%−16%, where DX is the dose delivered to X% of the structure volume and VX denotes the percentage of the structure volume receiving X% of the prescription dose. Plans were manually optimized and final source positions were a modified‐peripheral loading pattern. We routinely plan with a margin on the prostate, and consider seed placement just outside the prostate capsule to ensure adequate dose at the boundaries of the target volume. To limit the seeds placed outside the prostate, other statistics included in our guidelines are the percentage of seeds located in the target (>75%) and the percentage of seeds located in the gland (>50%). We independently double planned with two experienced planners, to reduce planner bias in our results. Dosimetry from the original preplans was then compared to the dosimetry from the replanned cases.

### B. Clinical study

Following the planning study, which showed a number of potential advantages for using higher strength seeds, a series of patients were implanted using seeds of strength ∼0.7U. A new series of 20 consecutive patients (as distinct from the planning study group of patients) was selected for this part of the study; 10 patients were implanted with 0.4 U seeds (standard strength) (median 0.388 U, range 0.310 U–0.399 U), and the next ten patients implanted with 0.7 U seeds (median 0.693 U, range 0.684 U–0.701 U). All PSI were performed between February and May 2005.

For all PSI patients, a volume study is obtained about six weeks prior to the implant, and individual plans (‘preplans’) are prepared. At the time of this study, typically a combination of loose seeds with spacer material and the stranded seed variety (RAPIDstrand; GE Healthcare, Waukesha, WI (formerly Amersham pic)) were used in the plan, and all needles were preloaded with the seeds several days before the implant. The seeds were implanted by a team of two, a radiation oncologist and an urologist in an operating room. Approximately one month (± one week) after their seed implant, patients returned to the WBRC for a CT scan from which seeds were localized and postplan dosimetry was calculated and analyzed. The exact time of the postimplant CT was determined by equipment, radiation oncologist, and patient availability. Postplan dosimetry for the target volume, urethra, and rectal wall was compared between the two groups of patients. Details of implant times were collected.

Clinical follow‐up data have been collected over six years, and include lower urinary tract symptoms recorded as the International Prostate Symptom Score (IPSS),^(15)^ RTOG rectal grade toxicity,^(16)^ and short International Index of Erectile Function (IIEF‐5).^(17)^ Data are collected from each patient postimplant at six‐month intervals for two years, and then annually.

## III. RESULTS

### A. Planning study

**Figure 1:**
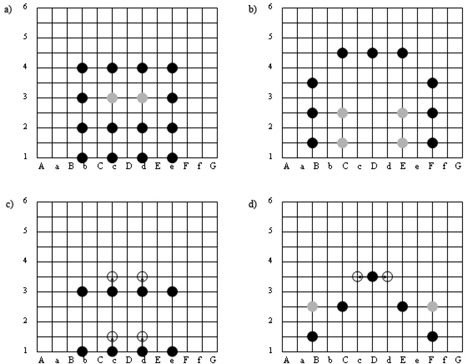
Typical seed arrangements on our planning template for 0.4 U plans (a) odd‐numbered and (b) even‐numbered planes, and 0.7 U plans (c) odd‐numbered and (d) even‐numbered planes. Black dots show seed positions, grey dots show positions of extra seeds added when necessary, and unfilled circles show alternative seed positions as indicated by arrows.

Differences in final seed loading patterns for the manually optimized plans from each seed strength group can be reported. Typically on the slice superior to base (in the region of the seminal vesicles), between six and seven seeds are planned using our standard seed strength of 0.4 U. When planning with higher strength seeds, the number of seeds on this slice was reduced to between two and six seeds. For the 0.7 U group, seed placement was typically around the periphery of the target on all slices, to avoid overdosing the urethra. Base and apex slices were almost fully loaded. As depicted in Fig. 1, on the remaining central slices, anterior and posterior coverage of the target was mostly from anterior and posterior rows of seeds on odd‐numbered planes, and lateral coverage mostly from lateral columns (or a pyramid‐type distribution) of seeds on even‐numbered planes.

The main results from the planning study are summarized in Table 1. Preplanning results shown are mean values and standard deviations (SDs) (in brackets) for the 12 plans, from the two planners. Satisfactory dosimetry, falling within our guidelines for the V100 (%), V150 (%), and D100 (Gy), could be obtained using seeds of any strength between 0.4 and 0.7 U. For the 0.6 U and 0.7 U plans, the upper range of the V200 (%) was approximately 22%. This is higher than current WBRC guidelines, but a V200≤25% is a typical preplanning limit in other centers.^18^, ^19^ For 0.6 U seeds, a reduction in the total number of seeds and needles used per implant was evident, which would have some advantages. However for 0.7 U seeds, with a more significant reduction in needle and seed numbers, this seed strength would provide the most practical benefits, such as an increase in target coverage (D100 (Gy)) and a reduction in the seed/needle loading time, theatre time, cost of seeds, and extent of patient tissue trauma.

Table 2 displays the D100 (Gy) and V100 (%) as a function of target volume size, for each seed strength investigated in this planning study. Although these dosimetry values are mostly higher for medium and large target volumes than small target volumes, there does not appear to be a definitive relationship between dosimetry, seed strength, and target volume. However, in going from 0.4 U to 0.7 U in these seed plans, the greatest percentage reduction in the number of seeds and needles was noted for the largest target volumes.

**Table 1:** Mean values from the preplanning study (SD)

	*0.4 U*	*Seed Strength 0.6 U*	*0.7 U*
D100 (Gy)	105.8 (7.4)	110.3 (5.6)	111.7 (8.9)
V100 (%)	98.0 (0.7)	98.4 (0.6)	98.5 (1.1)
V150 (%)	57.2 (2.6)	56.7 (2.5)	57.9 (3.5)
V200 (%) (range)	12.5‐14.9	16.1‐21.2	14.9‐21.5
Number of seeds^a^	112 (14)	78 (9)	68 (7)
Number of needles^a^	29 (3)	22 (3)	20 (2)

a Numbers rounded to nearest whole number.

**Table 2:** Mean dosimetry values (SD) and implant statistics (SD) for three seed strengths grouped according to target volume

*Seed Strength (U)*	*Small Target Volume*				*Medium Target Volume*				*Large Target Volume*			
	*V100 (%)*	*D100 (Gy)*	*Seeds* ^a^	*Needles* ^a^	*V100 (%)*	*D100 (Gy)*	*Seeds* ^a^	*Needles* ^a^	*V100 (%)*	*D100 (Gy)*	*Seeds* ^a^	*Needles* ^a^
0.4	97.9 (0.6)	103.7 (4.5)	94 (4)	26 (0)	98.1 (0.8)	108.3 (8.7)	116 (3)	29 (3)	98.0 (0.5)	105.4 (7.4)	125 (6)	31 (3)
0.6	98.6 (0.3)	108.6 (3.3)	67 (3)	20 (2)	98.5 (0.8)	113.8 (6.9)	82 (3)	23 (2)	98.5 (0.3)	110.9 (5.0)	85 (2)	23 (3)
0.7	98.1 (1.4)	105.5 (7.2)	59 (2)	19 (1)	98.7 (1.0)	115.4 (10.7)	69 (3)	21 (3)	98.8 (0.6)	114.2 (5.7)	76 (3)	19 (2)

a Numbers rounded to nearest whole number.

### B. Clinical study

**Figure 2:**
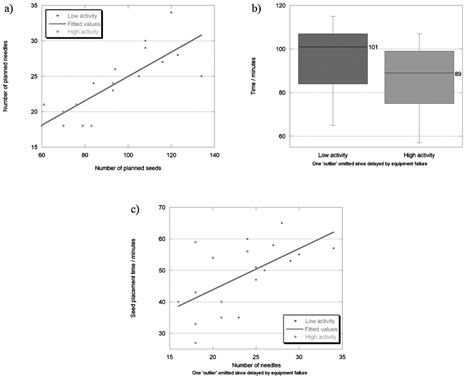
Implant details for the 20 plans in our clinical study showing: a) plot of the number of planned needles versus the number of planned seeds, b) total implant times related to seed activity, and c) plot of the seed implant time (seed placement time only, patient setup time excluded) vs. the number of implanted needles.

Figure 2(a) is a plot of the number of planned seeds versus the number of planned needles for the high (0.7 U) and low (0.4 U) activity implants. The mean number of seeds required per implant decreased by over 30% for the 0.7 U implants, and the mean number of needles decreased by eight needles. This led to a total time savings at implant of approximately 10 min (see Fig. 2(b)).

Figure 2(b) shows median values (bar), interquartile ranges (box), and adjacent values (whiskers). As determined from a plot of the number of needles versus seed placement time (Fig. 2(c)), each additional needle increases the seed implant time by approximately 1 min 46 s.

Preimplant and postimplant dosimetry parameters from the clinical study are displayed in Table 3. Mean postplan prostate dose coverage (as reflected in the D90 and D100 values) was slightly less for the 0.7 U group than the 0.4 U group, but all values were similar to within 5 Gy. However, these parameters were spread over a considerably wider range of values for the 0.7 U group, as seen in the SDs and ranges.

Mean postplan prostate dose homogeneity (as reflected in the V100, V150, and V200 values) was also slightly lower for the 0.7 U implants than the 0.4 U implants, but with differences of approximately ≤ 4%, these dosimetry values were not too dissimilar. The mean postplan V100 (%) was approximately 90% for both the 0.4 U and 0.7 U implants. For the higher activity implants there was a higher V100 (%) SD (mean difference in SD 6.2%). The mean postplan values of V150 (%) and V200 (%) are approximately 50% and 20%, respectively, for the 0.4 U and 0.7 U implants, with similar SDs for both seed strengths.

The mean urethral D10 (Gy) was 4.0 Gy lower for the 0.7 U group, and the mean rectal wall D2cc (Gy) was 15.7 Gy higher for 0.7 U group, but both seed strength groups shared similar ranges for these dosimetry parameters.

**Figure 3:**
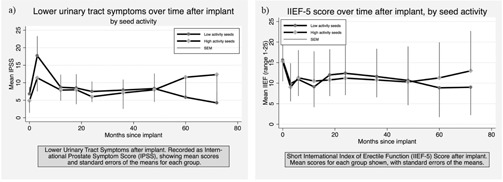
Clinical patient follow‐up data over time after implant for: a) lower urinary tract symptoms, and b) IIEF‐5 score.

Six‐year clinical follow‐up data from patients is shown in Fig. 3 and Table 4 for all patients alive^*^ and willing to submit follow‐up data. Although urinary symptoms at three months postimplant were greater for the 0.4 U group, and at five and six years they were greater for the 0.7 U group, considering the range of the standard error of the mean (SEM) there is no significant difference between the 0.4 U and 0.7 U groups. Table 4 shows rectal toxicity has been minimal for the two groups over the six years (mean scores<1), with the 0.4 U group recording slightly higher values. Overall the difference in rectal toxicity between the two groups has been insignificant. Similarly, the difference in erectile function between the two groups has also been insignificant from the time of the implant to follow up at six years (see Fig. 3(b)).

**Table 3:** Clinical study preplan and postimplant dosimetry results for the target volume, urethra, and rectal wall, and seed and needle statistics

*Seed Strength (U)*	*Preplan 0.4 U*		*Preplan 0.7 U*		*Postplan 0.4 U*		*Postplan 0.7 U*		*Postplan 0.7 U* ^b^	
	*Mean (SD)*	*Range*	*Mean (SD)*	*Range*	*Mean (SD)*	*Range*	*Mean (SD)*	*Range*	*Mean (SD)* ^b^	*Range* ^b^
D90 (Gy)	170.3 (13.5)	133.0‐181.9	175.3 (5.9)	159.8‐180.3	148.3 (14.7)	124.2‐175.7	143.4 (23.1)	101.3‐179.1	148.0 (18.8)	114‐179.1
D100 (Gy)	103.9 (10.5)	77.7‐117.2	110.7 (7.3)	93.5‐100.0	83.4 (11.0)	63.5‐99.1	80.1 (25.5)	40.6‐117.1	84.5 (22.7)	48.0‐117.1
V100 (%)	97.8 (0.5)	97.1‐98.3	98.0 (1.5)	94.2‐99.3	91.4 (4.1)	85.1‐96.7	88.3 (10.3)	63.8‐98.2	91.0 (6.1)	80.7‐98.2
V150 (%)	59.2 (1.5)	56.4‐61.1	57.9 (2.1)	54.8‐61.4	52.3 (13.2)	29.5‐72.6	48.2 (13.1)	27.9‐65.1	50.5 (11.8)	27.3‐65.1
V200 (%)	15.1 (0.9)	13.9‐16.6	18.5 (0.8)	17.5‐20.0	21.5 (7.6)	8.7‐34.4	19.5 (7.2)	9.2‐29.3	20.6 (6.8)	9.2‐29.3
Rectal wall D2cc (Gy)	99.1 (13.9)	78.2‐123.7	110.6 (15.6)	90.5‐139.7	107.5 (26.4)	75.5‐160.6	123.2 (30.7)	71.5‐161.0		
Urethra D10 (Gy)	203.4 (18.4)	155.2‐217.9	200.1 (9.4)	188.0‐213.4	219.9 (30.0)	176.8‐278.4	215.9 (37.2)	136.2‐278.4		
US target or CT prostate volume (cc)	56.5 (11.4)	39.2‐76.1	61.5 (15.7)	43.0‐95.0	32.3 (10.3)	23.1‐53.1	33.1 (5.4)	26.1‐43.1		
Mean number of seeds^a^	109 (15)	71 (12)						
Mean number of needles^a^	27 (3)	19 (2)						

a Numbers rounded to nearest whole number.

b Results excluding least desirable implant.

**Table 4:** Rectal RTOG grade toxicity over time after implant

*Seed Strength*	*RTOG grade*	*Number of Patients at Time‐Point*		
		*3 months*	*36 months*	*72 months*
	0	4	7	5
	1	4	3	3
0.4 U	2	2	0	0
	3 to 5	0	0	0
	Mean score	0.8	0.3	0.4
	0	6	7	6
	1	4	2	2
0.7 U	2	0	0	0
	3 to 5	0	0	0
	Mean score	0.4	0.2	0.3

## IV. DISCUSSION

This study has shown that acceptable implants can be achieved for our I‐125 prostate implants using a range of seed strengths between 0.4 U and 0.7 U and manual‐optimized planning techniques. Our planning study and the literature^2^, ^3^, ^4^, ^6^, ^7^, ^8^, ^9^, ^11^ suggested this would be the case, and in our clinical study the resultant calculated mean dosimetry was only marginally different between our patient groups. The mean values of dose coverage and dose homogeneity for the 0.7 U seed strength implants were slightly lower than the 0.4 U seed strength implants. The preplanning study and the actual preplanned values for the clinical study patients would suggest a higher D100 (%) and more hot spots (a higher V200) could be expected for the higher seed strength implants. Deviations in the implant dosimetry from the preplan dosimetry were most probably due to seed migration, seed placement error, and/or prostate edema effects. This study was limited to two experienced radiation oncologists to reduce uncertainty and ensure consistency in contouring planning target volumes and postimplant CT prostate volumes. Postimplant CT scans at the WBRC were performed at one month (± one week) following the seed implant, to provide consistency in recording postimplant dosimetry. Retrospectively, this is the recommended time interval for I‐125 seeds, according to TG 137,^(20)^ to minimize prostate edema effects in postimplant dosimetry. However at this time interval, it is not possible to eliminate the anisotropic edema characteristics specific to individual patients. If prostate edema was present at the time of the CT scan, then postimplant dosimetry is more likely to be underestimated for those patients, and this effect cannot be excluded from the clinical dosimetry results of both groups of implants in this study, as is usually the case when postimplant dosimetry is done in conformance with AAPM guidelines.

Standard deviations in the postimplant D90 (Gy), D100 (Gy), and V100 (%) were larger for the 0.7 U group, and data ranges for all dosimetry parameters, except the V200, were more widely spread for this group, too. While notably larger data ranges were not predicted from our preplanning study or our preplanned clinical dosimetry values, greater sensitivity of the higher seed strength plans to source displacement was indicated in other studies.^5^, ^10^ In both of these studies, the planning technique is considerably different to that at WBRC. For instance, we routinely plan with a margin on the prostate, and consider seed placement just outside the prostate capsule (but usually within the target volume). As the effects of seed placement errors would be extremely dependent on the planning method, it is difficult to directly apply the results of other studies to our plans without further modeling. However it is most likely that seed placement error or migration is responsible for the larger SDs and ranges we observed for our 0.7 U implants. Our “action level” for an undesirable implant is a D90 of 100 Gy, and additional treatment with external beam radiotherapy would be considered for such an implant. A single implant in the 0.7 U group approached this threshold. Excluding this nonideal implant from our dosimetry results gives mean postplan dosimetry values similar to the 0.4 U group and somewhat reduces the differences in SDs and ranges of these values (see final columns in Table 3).

To minimize seed migration and the likelihood of an undesirable implant for 0.7 U implants containing fewer numbers of sources, we recommend a thorough consideration of the percentage of seeds planned within the prostate volume. Our current preplanning guidelines ensuring >50% of seeds are located within the gland are not adequate for 0.7 U implants, and would be increased (to at least >75%) before additional implants were considered at WBRC.

Urethral doses were acceptably low for the 0.4 U and 0.7 U implants, and urinary tract symptoms over time have not been too affected by using a higher seed strength. Urethral sparing was easy to achieve with 0.7 U seeds, as preplanning limits exist for urethral doses, and the 0.7 U plans were more “peripheral” than the 0.4 U plans. The modified‐peripheral plans most likely resulted in the low urethral doses with the higher strength seeds.

Rectal wall doses, although not exceedingly high, have been increased for the 0.7 U group. Although this result is not alarming and did not lead to long‐term rectal toxicity, it is not desirable either. Patients in the two groups were selected as consecutive treatments and not matched according to target size. Mean ultrasound target volumes were marginally higher for the 0.7 U group, and correspondingly there was a higher mean total implanted AKR for this group (49% for 0.7 U compared with 42% for 0.4 U). Higher rectal wall doses may be a result of the higher mean total implanted AKR for the higher seed strength group. Even though we did not see a higher incidence of late‐rectal toxicities, as was observed by Usamani et al.,^(9)^ more consideration of this critical organ is possible at the preplanning stage for 0.7 U implants via dose constraints and considering source placement along the posterior edge of the gland and the target in the planning method. A delicate balance of urethral and rectal doses in relation to source placement is essential,^(13)^ but for some implants, it may be possible to increase the spacing between the posterior plane of seeds and the posterior border of the prostate beyond the 5 mm as suggested by Butler et al.^(12)^ A preplanning dose limit for this organ, and less emphasis on coverage of the target with the 100% isodose in this region, would also reduce rectal wall dose.

There are some practical advantages of using higher strength seeds for prostate implants. In particular, for 0.7 U seed implants there is a substantial reduction in the total number of needles used compared with 0.4 U implants, which decreases the theatre time and associated costs required for the implant. At WBRC we typically implant two patients per theatre session, and a ∼10 min time saving per implant would not permit us to implant an extra patient per theatre session. However, this level of time savings would benefit centers with multiple (>2) implants per day. Additionally, a 30% reduction in the number of needles per implant for 0.7 U seeds compared to 0.4 U seeds would generate a significant time savings in needle loading time for higher seed strength implants. At the time of this study, our needles were loaded by a physicist prior to the implant. Although we did not collect data on time taken for our needle loading for the 20 patients in this study, we believe that loading 8–10 fewer needles per implant (with 0.7 U seeds) would benefit the physicist with time constraints who loads needles in theatre and/or immediately prior to the implant. The extent of patient tissue trauma resulting from fewer number of needle sticks was not investigated in this particular study.

Our clinical study was relatively small and, as such, might not provide reliable estimates of average doses. Nonetheless, we are confident that the results seen in the postimplant dosimetry after high activity implants are consistent with the findings of our planning study, that similar dose distributions could be achieved without an undue risk of high‐ or low‐dose volumes in the prostate target volume.

## V. CONCLUSIONS

We have shown in a planning and clinical study that prostate seed implants using higher strength seeds of 0.7 U with manual planning and optimization methods, produced satisfactory implants. Our six‐year clinical follow‐up data show promising results, with no increase in urinary, erectile or rectal complications for patients implanted with the higher strength seeds. For centers with similar planning techniques to WBRC, we have demonstrated potential changes to the preplan dosimetry that can be expected for 0.7 U seed strengths, and the importance of a thorough consideration of the number of seeds implanted within the gland, as well as seed positions relative to urethral and rectal sparing. We will consider the use of 0.7 U seeds for patients with large prostate volumes at WBRC, where a majority of the seeds can be placed within the prostate capsule.

## ACKNOWLEDGMENTS

The authors wish to thank the WBRC Brachytherapy team for their contributions to this study.
